# Exploring a Structural Model of Teaching Enjoyment, Teacher Self-Efficacy, and Work Engagement

**DOI:** 10.3389/fpsyg.2022.918488

**Published:** 2022-06-16

**Authors:** Yan Xiao, Jalil Fathi, Farnoosh Mohammaddokht

**Affiliations:** ^1^Shanghai Customs College, Shanghai, China; ^2^Faculty of Language and Literature, Department of English and Linguistics, University of Kurdistan, Sanandaj, Iran

**Keywords:** teacher self-efficacy, teaching enjoyment, work engagement, EFL teachers, CFA

## Abstract

Due to the complexity of teaching, determining the variables influencing teachers’ work engagement is a rewarding research area. In line with this research agenda, the purpose of this study was to test a structural model of work engagement based on teacher self-efficacy and teaching enjoyment among English as a foreign language (EFL) teachers. For this purpose, 315 Iranian English instructors completed an online survey. Initially, the measurement models for the three latent variables were averred *via* conducting confirmatory factor analysis (CFA). Following that, structural equation modeling (SEM) was utilized to test the hypothesized model. SEM results showed that both self-efficacy and teaching enjoyment were the significant predictors of teachers’ work engagement, although teacher self-efficacy was a stronger predictor than teaching enjoyment. The findings might have notable implications for English teachers.

## Introduction

With the advent of positive psychology in second language (L2) learning, researchers have become increasingly interested in the psychology of language and made attempts to add novel approaches to the language teaching field (MacIntyre et al., 2016). A central mission of positive psychology which was first introduced in the late 1990s (see Seligman and Csikszentmihalyi, 2000) is to identify, develop, and evaluate interventions that aim to increase wellbeing among people. At a broad level, positive psychology theory and research have focused on imaging one’s best possible self, building resilience as well as wellbeing, and other related positive concepts (Seligman and Csikszentmihalyi, 2000; Seligman, 2002). Over the past two decades, this branch of psychology has experienced rapid growth in studies investigating the concept of emotions, processes, strengths that build optimal functioning and foster furnishing (Seligman and Csikszentmihalyi, 2000; Fredrickson, 2001) in teachers. Therefore, with the turn of the millennium, researchers have grasped a more nuanced understanding of the effect of teacher emotions by conducting various methodological approaches. More light is starting to be shed on teachers’ mental health and wellbeing in foreign language (FL) teaching (Mercer and Gregersen, 2020) since teachers are now widely recognized as the central component of educational system (Derakhshan et al., 2020; Gao and Zhang, 2020).

Due to the significance of emotional wellbeing as well as the burgeoning of positive psychology both in the field of psychology (e.g., Seligman and Csikszentmihalyi, 2000; Fredrickson, 2009) and FL education (MacIntyre and Gregersen, 2012; Dewaele et al., 2019a; Fathi and Mohammaddokht, 2021; Wang et al., 2021; Derakhshan et al., 2022), research has witnessed a surge of interest in teachers’ positive emotions (Cowie, 2011; Khajavy, 2021). Hence, there has been an outstanding shift from exploring negative variables, including teacher stress (Fathi and Derakhshan, 2019) and burnout (Safari, 2021) to examining positive factors such as work engagement (Dong and Xu, 2022), enjoyment (Ergün and Dewaele, 2021), emotion regulation (Fathi et al., 2021a), psychological well-being (Greenier et al., 2021), resilience (Ayoobiyan and Rashidi, 2021), and job satisfaction (Safari, 2021) and other emotion variables (Cowie, 2011; Khajavy et al., 2018) in FL contexts. According to Boudreau et al. (2018), FL enjoyment (FLE) as a positive emotion has sparked many researchers’ keen interest in recent years (Dewaele and Li, 2022). FLE is defined as a quite stable and complex emotion which is distinguished from the superficial experience of euphoria. However, multiple studies have highlighted the dynamic, multifaceted, and complex nature of FLE (Dewaele and Dewaele, 2017; Elahi Shirvan and Talebzadeh, 2018).

As Bandura’s (1997) self-efficacy construct became more known, researchers started to notice the important concept of teacher self-efficacy (Choi et al., 2019). Notably, domain-specific teachers’ self-efficacy is conceptualized as individual teachers’ judgment of their capabilities to bring about optimal outcomes of learning and student engagement (Tschannen-Moran and Woolfolk Hoy, 2001; Choi et al., 2019). Simply put, teacher self-efficacy is defined as teacher’s perceptions toward his or her abilities to be an effective teacher (Morris et al., 2017). In this respect, accumulated evidence shows that teacher self-efficacy is regarded as a key determinant of teacher job satisfaction (e.g., Klassen and Chiu, 2010). In addition, researchers have also underscored the effect of teacher self-efficacy on teacher engagement (Skaalvik and Skaalvik, 2014, 2019), students’ academic adjustment, patterns of teachers’ behavior, and teachers’ psychological well-being (e.g., Zee and Koomen, 2016).

As another variable under investigation in this study, work engagement, a state of enthusiasm, energy, motivation caused by work, is regarded as a work-related wellbeing dimension (Rothmann, 2008) and, in particular, has been contributed to physical and psychological health at work (Bao et al., 2021; Greenier et al., 2021). Work engagement is concerned with how individuals put energy and time into accomplishing a task which is affected by different external and internal variables (Han and Wang, 2021). Given the turnover of many teachers in some modern education systems (Perera et al., 2018a), policy-makers and researchers have become interested in examining teacher work engagement (Perera et al., 2018b; Granziera and Perera, 2019), hoping to find the reasons why teachers leave their profession. Even though a bulk of studies were previously conducted on job burnout among L2 language teachers (Khajavy et al., 2017), there is a dearth of research on English as a foreign language (EFL) teachers’ engagement which has created a need for further studies in this field to fill the current research lacuna. At the same time, whereas a bulk of research has been done on each variable of teacher self-efficacy and work engagement in general education, no study has explored their relation to teacher enjoyment simultaneously in EFL contexts. As an attempt to fill the existing research gap, the present study examined three variables of teacher self-efficacy, foreign language teaching enjoyment (FLTE), and work engagement of Iranian EFL teachers. Accordingly, the major research question in the current study undertaking is whether and how these factors are associated.

Given the findings reported in the literature (e.g., Skaalvik and Skaalvik, 2014, 2017; Burić and Macuka, 2018; Minghui et al., 2018; Han and Wang, 2021), we hypothesize that teachers’ self-efficacy positively affects their work engagement. Also, in light of the findings of empirical studies regarding the association between positive emotions and work engagement (e.g., Ouweneel et al., 2012; Gloria and Steinhardt, 2017; Burić and Macuka, 2018), we hypothesize that teaching enjoyment positively influences teacher work engagement.

## Literature Review

### Foreign Language Teaching Enjoyment

After the advent of positive psychology as a well-established discipline of psychology aiming to show a better understanding of the concepts that make individuals flourish and thrive (Seligman and Csikszentmihalyi, 2000), many scholars were encouraged to hold a holistic perspective toward emotions (e.g., Dewaele and MacIntyre, 2014; MacIntyre and Vincze, 2017; Dewaele et al., 2018). Gradually, interest grew in positive emotions (e.g., Dewaele et al., 2018) as they could contribute to learners’ wellbeing and academic achievement (Li and Xu, 2019). Since the focus was mostly on negative factors in the past, there was a lack of investigation into the positive side. Stimulated by the positive psychology development during recent years, FL scholars have currently begun to maintain a balance between the assessments of both negative and positive emotions (e.g., Liu and Wang, 2021). From a holistic perspective, positive and negative emotions integrated together as fuel would drive language learning forward (MacIntyre and Vincze, 2017); Fredrickson (1998, 2001)) *Broaden and Build Theory* has advanced our knowledge of positive emotions, suggesting that positive emotions result in both well-being and prosperity in life. Positive emotions, according to this theory (Fredrickson, 1998, 2013), aid learners in expanding their thought-action repertoire as well as forming the intellectual, physical, psychological, and social resources required for their prospective development. Similar to negative emotions (e.g., anxiety and boredom), learners also experience some positive emotions (e.g., a sense of achievement and enjoyment) in their language learning process (MacIntyre and Vincze, 2017). Pleasant emotions have enduring consequences in the classroom since they create enjoyable moments for learners to feel happier, which in turn lead to increased hardiness and resilience in inevitable difficult times (Prior, 2019). Also, MacIntyre and Gregersen (2012) argued, positive emotions increase not only students’ competence in noticing different things in the classroom but also their perception of language input. As a result, awareness of positive emotions helps learners maintain and develop their perseverance and motivation for their language learning journey. Moreover, teachers will recognize the salient role of positive emotions and take initiatives to create a good emotional climate. In this respect, several researchers have supported the fact that high-caring, positive, and friendly classroom environment with higher peer-interactions increases EFL learners’ enjoyment (Elahi Shirvan and Taherian, 2020).

Foreign language enjoyment, so far, has become one of the most investigated positive emotions (see Jiang and Dewaele, 2019) in various contexts. Dewaele and MacIntyre (2016) conceptualized FLE in language learning as learners’ efforts in the classroom to overcome learning obstacles and develop their proficiency and knowledge. The findings of Dewaele et al. (2018) showed that FLE was more correlated with learner internal factors such as gender, attitudes, FL proficiency levels, and age than teacher-centered factors. Furthermore, female learners reported more FLE. This is confirmed by Dewaele et al. (2016) who showed that female learners had higher levels of excitement and enjoyment than males in the FL classroom. In another study, Jiang and Dewaele (2019) verified the stronger effect of teacher variables such as teacher’s joking, friendliness, strictness, and unpredictability in increasing FLE. Regarding teacher characteristics, Dewaele and Dewaele (2020) and Dewaele et al. (2019b) underscored the influence of teachers’ use of FL in boosting learners’ FLE. However, Dewaele et al.’s (2019b) study showed that teachers’ foreign accent was a negative predictor of FLE. In order to test the psychometric qualities of FLE scales across time, Elahi Shirvan et al. (2021) conducted a longitudinal confirmatory factor analysis (CFA) alongside the developments in FLE measurement. They also revealed that those language learners who had higher FLE did not report great changes in this concept across time, whereas learners with lower degrees of FLE experienced remarkable alterations in the long run. In another study, Dewaele and Li (2022) made an attempt to uncover the relation of FLE and FL classroom anxiety (FLCA) with overall FL achievement as well as self-perceived achievement within six areas across speaking, listening, writing, reading, grammar, and vocabulary. The findings suggested that FLCA and FLE were associated with more self-perceived general English proficiency, and less actual English achievement. Moreover, FLE was significantly predicted by perceived speaking, reading, and grammar competence. Nevertheless, FLE and FLCA were not predicted by perceived writing, listening, and vocabulary competence.

Grounded in the general notion of FLE, FLTE generally refers to teachers’ joy, pleasure and happiness in teaching a FL (Mierzwa, 2019). As one of the leading studies, Ergün and Dewaele (2021) conceptualized FLTE as a three-dimension construct constituting (a) teachers’ ability in providing a pleasant classroom atmosphere, (b) teachers’ personal enjoyment of learning a FL, and (c) social pleasure associated with FL, which refers to social solidarity with peers. Mierzwa (2019) also probed into the concept of FL teaching enjoyment (FLTE). The results of this study demonstrated that teacher specific factors such as years of teaching experience, gender, the language teachers teach, and place of residence were not predictors of teachers’ enjoyment. Likewise, Ergün and Dewaele (2021) examined the construct of FLTE with a sample of 174 Italian FL teachers. For doing so, they employed the FLTE developed by Botes et al. (2021). Their findings reported that although both teacher resilience and wellbeing predicted FLTE, resilience was a stronger predictor. They also posited that FL teachers with higher FLTE experience higher levels of happiness, job satisfaction, and lower degrees of happiness. Put it differently, these teachers tend to feel less work burnout and exhaustion.

### Teacher Self-Efficacy

As illustrated by Bandura (1997), self-efficacy pertains to one’s belief regarding their own capability to perform specific tasks effectively. The first studies on teacher self-efficacy were done with the Rand Corporation in the late 1970s (Armor et al., 1976), which were based on the seminal work of Rotter (1966) and, to a greater extent, Bandura’s (1997) social cognitive theory. Drawing on Bandura’s (1997) social cognitive theory, teacher self-efficacy is conceptualized as teachers’ beliefs in their own capabilities to perform particular teaching tasks at a particular level of quality in a specific situation. Ever since the work of the Rand research in the 1970s (Armor et al., 1976), a mounting body of research has measured teacher self-efficacy (e.g., Tschannen-Moran and Woolfolk Hoy, 2001) and its relationship with various constructs. Studies on teachers’ characteristics have demonstrated that self-efficacy is positively correlated with job satisfaction (Skaalvik and Skaalvik, 2014), work engagement (Minghui et al., 2018; Han and Wang, 2021), organizational commitment (Waweru et al., 2021), and negatively linked to burnout (Skaalvik and Skaalvik, 2007, 2014; Fathi et al., 2021a). For instance, the findings obtained from Skaalvik and Skaalvik (2014) among 2,569 instructors in schools indicated that teachers who have an assured sense of self-efficacy experience greater job satisfaction and are less emotionally exhausted. Likewise, Fathi et al. (2021a) revealed that job burnout and teacher self-efficacy were negatively correlated. Additionally, teachers with high self-efficacy are better able to cooperate with their colleagues in terms of shared educational aims (Goddard and Kim, 2018) and perceive less student misbehavior (Tsouloupas et al., 2010). Using a variety of measures and definitions, research implies that efficacious teachers create a high-quality classroom climate by planning engaging lessons that advance learners’ abilities, by effectively managing student misbehaviors, and by attempting to engage students in a meaningful way (Tsouloupas et al., 2010). It is widely agreed that teachers with greater sense of self-efficacy set the tone to have closer relationships with their students and interact in a way that fosters students’ behavioral functioning (Hamre et al., 2008).

With a sample of Croatian teachers, Burić and Macuka (2018) showed that teachers who had high self-efficacy reported more engagement in their work, more pride, love, and joy, and less fatigue, hopelessness, and anger toward their learners. Language teacher self-efficacy is regarded as a developing research field, which has been measured in a few East Asian contexts (e.g., Phan and Locke, 2015). However, the investigation of EFL teacher self-efficacy is a relatively recent branch of research (see Hoang and Wyatt, 2021). Using a mixed-methods approach, Choi and Lee (2018) examined the association between teacher self-efficacy and teaching practices among 190 EFL practitioners. Findings revealed a substantial association between overall self-efficacy and the use of teaching practice. Moreover, the results from the interviews indicated that some beliefs regarding ideal ways of English instruction as well as sociocultural variables affected the link between actual teaching and efficacy beliefs. In a subsequent study, Hoang and Wyatt (2021) highlighted the pivotal role of culture and context in shaping Vietnamese pre-service teachers’ self-efficacy beliefs, their instructional strategies, classroom management, and student misbehavior management. In another study, Fathi et al. (2021b) examined two types of teacher efficacy (i.e., individual self-efficacy and collective efficacy) and their relation to work engagement among Iranian EFL teachers. Teachers’ individual self-efficacy was reported to be a more powerful contributor of work engagement than collective efficacy. Conducting a structural equation modeling (SEM) of self-efficacy, emotion regulation, burnout, and teacher reflection among EFL teachers, Fathi et al. (2021a) found a positive correlation between teacher self-efficacy and emotion regulation. Also, among all the variables (i.e., institutional identity, critical cultural awareness, reflective teaching, and self-efficacy) that Soodmand Afshar and Moradifar (2021) used in their study, self-efficacy was the strongest predictor of job performance among Iranian EFL teachers.

### Work Engagement

Work engagement, in recent years, has become a popular concept and a subject of ever-increasing interest in the domain of positive psychology and management during the past decade (Liu et al., 2020). Work engagement was originally conceptualized in terms of individuals, particularly employees being cognitively, emotionally, and physically involved in their role performances. Since then, Schaufeli et al.’s (2002) conceptualization of work engagement as involving absorption (being immersed and having full concentration at work), vigor (mental resilience, persistence, willingness, and energy at work), and dedication (enthusiasm, involvement, and inspiration at work) has become the most accepted and prevalent definition (see Hakanan and Roodt, 2010). Work engagement is not a specific and momentary state of mind, but a more pervasive and persistent cognitive state which is not related to a particular event, individual, behavior, or object (Schaufeli et al., 2002). The work engagement notion is grounded in the theory of work engagement, highlighting the importance of enthusiasm, vitality, and personal enjoyment, which drive individuals forward in their job performance (Han and Wang, 2021).

Work engagement concept emerged from burnout investigation, aiming to focus on employee’s well-being and ways to enhance it rather than their degree of burnout (Zeng et al., 2019). Recently, work engagement as a positive opponent of job burnout, has been the concern of a myriad of studies (Juliana et al., 2021). Research has averred that work engagement is in a negative correlation with burnout and employees’ intention to quit (Maslach and Leiter, 1997; Schaufeli and Bakker, 2004; Demerouti et al., 2010; Juliana et al., 2021). This concept is believed to be characterized by individuals’ strong identification with their work and high levels of energy, while burnout is agreed to be characterized by the opposite counterpart: individuals’ poor identification with their work and low levels of energy (Demerouti et al., 2010). Maslach and Leiter (1997) also maintained that engagement is identified by efficacy, involvement, and energy, which are the opposites of the three dimensions of burnout. In the burnout case, efficacy turns into ineffectiveness, involvement into cynicism, and energy into exhaustion. As far as teaching is concerned, few investigations have probed into the association between teachers’ work engagement and positive emotions (Zeng et al., 2019; Greenier et al., 2021). Zeng et al. (2019) reported that incremental mindset and well-being act as the mediator factors in the relationship between work engagement and enjoyment. It was also revealed that teachers with growth mindset experienced enjoyable challenges. On the other hand, empirical evidence has suggested that higher work engagement results in lower job burnout (Faskhodi and Siyyari, 2018; Juliana et al., 2021). Also, teacher engagement has been investigated from the standpoint of length of teaching experience, teacher status, and gender. For instance, Topchyan and Woehler (2021) indicated that full-time teachers reported significantly higher global job engagement and more job satisfaction than substitute teachers. However, work engagement and job satisfaction were not associated with years of teaching experience. On the contrary, Faskhodi and Siyyari (2018) showed that Iranian EFL teachers’ higher teaching experience was significantly correlated with greater work engagement. From an online teaching perspective, Obrad and Circa (2021) revealed that perceived learner engagement as well as motivation were major determinants of teaching engagement. In another study, Zou et al. (2021) explored EFL writing instructors’ engagement with respect to online formative assessment. The results showed integral, auxiliary, and disturbing types of engagement as the three kinds of teacher engagement which were mediated by both technological and contextual factors. Teachers’ beliefs, teaching/learning experiences, and digital literacies were the predictors of the identified teacher engagements. In FL/L2 contexts, studies have found that engaged teachers have higher levels of energy, devote substantial cognitive repertoire to their work, and stay persistent in spite of facing the setbacks (Greenier et al., 2021). This reechoes with the theoretical evidence supporting the fact that that involvement, attention, and energy are the main components of work engagement (see Bakker et al., 2011).

Conducting a cross-cultural study, Greenier et al. (2021) reveled that psychological well-being and emotion regulation were significant predictors of both Iranian and British English instructors’ degree of engagement. Within this line of research, Xie (2021) utilized a mixed-methods design to probe the predictive role of resilience and emotion regulation (cognitive reappraisal and expressive suppression) among Chinese EFL teachers. The findings highlighted a moderate relationship between work engagement and cognitive reappraisal. Nonetheless, work engagement was not predicted by expressive suppression. Furthermore, resilience significantly predicted work engagement. Semi-structured interviews also reported extrinsic variables (e.g., close relationships with administrators/colleagues, high degree of support, students’ high degrees of achievement, engagement, and motivation) and intrinsic factors (sense of responsibility and accomplishment, and resilience at work) as contributors to work engagement of EFL teachers.

Although there is a considerable amount of research regarding EFL learners’ engagement (Dewaele and Li, 2021), scarce attention has been given to teachers’ work engagement in FL contexts. To support this claim, the reviewed literature suggests that there has been no study investigating the three factors of teacher self-efficacy, teaching enjoyment, and work engagement in EFL contexts. Accordingly, to contribute to the existing knowledge of EFL teacher education, this study sought to enlighten the significance of psychological variables of teachers. In particular, the current research aimed to investigate the structural relationships among teacher self-efficacy, FLTE, and work engagement among EFL instructors *via* adopting SEM.

## Materials and Methods

### Participants

A sample of 315 Iranian EFL instructors were recruited as the participants of this research. The sample included English teachers from Northern and Western cities and provinces in Iran. The teachers were selected *via* convenience sampling and included both male (*N* = 143) and female (*N* = 172) EFL practitioners who volunteered to take part in this research by filling out the online questionnaires. Participants’ ages ranged from 21 to 43 (*M* = 24.18, SD = 6.59) and their teaching experience varied from 10 months to 24 years (*M* = 4.88, SD = 1.95). Most of the participants were coming from English major backgrounds and had studied English in universities. All of the sample members were engaged in teaching EFL in public schools, institutions, or universities.

### Instruments

#### Teacher Self-Efficacy Scale

In this research, teachers’ degree of self-efficacy was assessed by the Teachers’ Sense of Efficacy Scale (TSES) constructed by Tschannen-Moran and Woolfolk Hoy (2001). This self-report scale includes 24 items which assess instructors’ attitudes toward their ability in using proper strategies, engaging their pupils, and classroom management. Every item is assessed on a 5-point Likert scale ranging from 1, “nothing,” to 5, “a great deal.”

#### Foreign Language Teaching Enjoyment Scale

In order to measure teaching enjoyment of the participants, the Foreign Language Teaching Enjoyment Scale (FLTES), validated by Ergün and Dewaele (2021), was employed and given to the participants. This scale includes 9 items which measure three underlying components: Personal Enjoyment (PE), Student Appreciation (SA), and Social Enjoyment (SE). Every item is measured on a 5-point Likert scale varying from 1 (strongly disagree) to 5 (strongly agree).

#### Teacher Work Engagement Scale

In the present study, teachers’ work engagement was measured using a self-report scale designed by Schaufeli et al. (2002). This scale measures respondents’ degree of work engagement in terms of three aspects: Vigor (VI), Dedication (DE), and Absorption (AB). Every item is assessed on a 7-point Likert scale varying from 0 (“never”) to 6 (“always”).

### Procedure

The purpose of this non-experimental study was to test a structural model pertaining to teacher variables. To this end, the sample who volunteered to take part in the research were asked to complete an online survey containing a battery of questionnaires measuring teacher self-efficacy, teaching enjoyment, and teacher work engagement. The online survey was created using the Google Docs application and the link of the electronic survey was shared with English instructors from different parts of the country. Most of the participants were members of online channels or groups in Telegram or WhatsApp in which the link was shared. Prior to answering the items of the self-report scales, the volunteer teachers were given explanations on how to complete the Google Docs forms. They were also informed that their provided information would be utilized for research purposes and remain confidential.

### Data Analysis

Statistical Package for Social Sciences (SPSS 22) and Analysis of Moment Structures (AMOS 21) software were utilized for the data analysis. In addition to calculating descriptive statistics, CFAs and Structural Equation Modelling (SEM) were also carried out. SEM, as a powerful multivariate technique, was used to confirm the suggested structural relations. CFA was run to examine the validity of the latent variables before investigating the structural model (Hair et al., 1998).

Missing data, outliers, and normality check were examined during an initial data screening. Missing data were examined using an expectation– maximization algorithm in which the missing data were replaced by random values (Kline, 2011). Univariate and multivariate outliers were examined through standard scores and Mahalanobis *D*^2^, respectively. Additionally, the kurtosis and skewness values falling outside the range of −1 to +1 were regarded non-normal. After the initial screening, all the outliers and non-normal data were detected and eliminated, leading to 269 valid cases for analyses.

The measurement models for the three latent constructs were examined through running CFAs and fit indices were considered to verify their validity (Kline, 2011). More particularly, Chi-square divided by degree of freedom (χ^2^/df), Comparative Fit Index (CFI), Tucker–Lewis Index (TLI), and Root Mean Square Error of Approximation (RMSEA). Following Tseng and Schmitt (2008), we regarded a model to be fit in case χ^2^/df < 3, CFI and TLI > 0.90, and RMSEA < 0.08.

## Results

As discussed before, the construct validity of the scales was first examined. The measurement models demonstrated good fit (see Table 1). As for the reliability coefficients, all the computed coefficient alphas for the measures were above 0.70, indicating the appropriateness of the internal consistencies (see Table 1). Afterward, descriptive statistics and correlation coefficients of the constructs were computed (see Table 2).

**TABLE 1:** Measurement model of the latent constructs.

	χ^2^	df	χ^2^/df	CFI	TLI	RMSEA	α
Self-efficacy	46.28	24	1.92	0.95	0.94	0.05	0.83
Teaching enjoyment	15.85	8	1.98	0.93	0.92	0.06	0.78
Engagement	8.92	5	1.78	0.98	0.97	0.04	0.91

**TABLE 2:** Descriptive statistics and correlations.

	*M* (SD)	1	2	3
(1) Self-efficacy	3.95 (1.03)	1.00		
(2) Teaching enjoyment	3.17 (0.89)	0.24*	1.00	
(3) Engagement	3.65 (0.96)	0.51**	0.38**	1.00

*p < 0.05; **p < 0.01.

### Structural Equation Modeling Analysis

**FIGURE 1:**
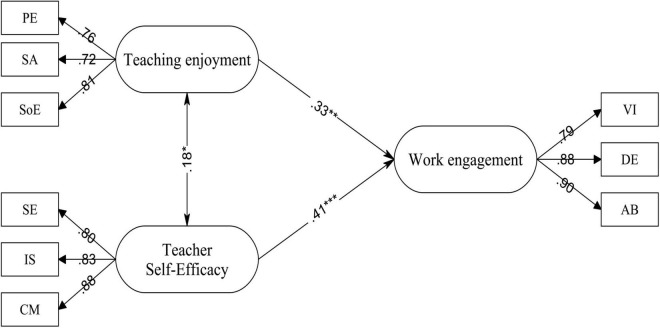
The final model of teacher self-efficacy, teaching enjoyment, and teacher work engagement. *p < 0.05; **p < 0.01; ***p < 0.001.

In the follow-up analysis, the structural model was evaluated employing AMOS 21 with the maximum likelihood procedure and variance-covariance matrices as the input. The outcomes showed that the path coefficients were significant (*p* < 0.05) and the fit indices were acceptable. SEM results verified the hypotheses in the proposed model (see Figure 1). Concerning the meaningfulness of data interpretations, effect size (ES) (Cohen’s *f*^2^) was calculated for the latent variables (Table 3).

**TABLE 3:** Standardized parameter estimates for the structural model.

	*R* ^2^	*f* ^2^
(1) Self-efficacy	0.16	0.20
(2) Teaching enjoyment	0.10	0.12

As depicted in Figure 1, teaching enjoyment exerted a small effect on teachers’ work engagement (β = 0.33, *R*^2^ = 0.10, *f*^2^ = 0.12, small ES). Furthermore, it was revealed that teacher self-efficacy was a more powerful correlate of teacher engagement (β = 0.41, *R*^2^ = 0.16, *f*^2^ = 0.20, medium ES).

## Discussion

To broaden the scope of research exploring teacher-related factors, this research aimed to examine a structural model of Iranian EFL teachers’ self-efficacy, teaching enjoyment, and work engagement. The findings from the structural model examination demonstrated that teacher self-efficacy was a substantial predictor of teacher work engagement. This finding resonates other studies (Skaalvik and Skaalvik, 2014, 2017; Burić and Macuka, 2018), highlighting that more efficacious instructors tend to have greater degrees of work engagement. For instance, Burić and Macuka (2018) demonstrated that self-efficacy was positively correlated with work engagement. This also concurs with Minghui et al. (2018) and Han and Wang (2021) who reported a significant association between teacher efficacy and work engagement among teachers. Likewise, Granziera and Perera (2019) reported that work engagement and teacher self-efficacy were reciprocally correlated over time. This result also verifies that of Topchyan and Woehler (2021) which revealed that self-efficacy positively predicts the cognitive work engagement of teachers. In a broad sense, people with higher efficacy perceptions tend to have a better performance at work as they are more likely to be persistent as well as diligent and experience lower degrees of anxiety (Bandura, 1997). The reason might lie in Bandura’s (1997) social cognitive theory. Since EFL teachers might be confident of their instructional expertise and capabilities in meeting learners’ needs, engaging learners, and running a fruitful course, they would become intrinsically motivated and keen to invest more effort and energy in accomplishing their pedagogic activities. As Tschannen-Moran and Woolfolk Hoy (2001) maintained, employees’ competence influences their motivation, perception, and performance.

Furthermore, it was revealed that FLTE could substantially predict teacher work engagement. This finding is consistent with other studies (Ouweneel et al., 2012; Gloria and Steinhardt, 2017; Burić and Macuka, 2018), which evidenced that positive emotions (joy and enjoyment) are predictors of work engagement. It might be stated that EFL teachers with high levels of FLTE are less prone to emotional exhaustion, job burnout, and hopelessness. Therefore, they become more enthusiastic, optimistic, and emotionally attached to their job since they can overcome stressors more easily and effectively. This is supported by Faskhodi and Siyyari (2018), suggesting that as the level of job burnout decreases, instructors’ work engagement increases. Likewise, Burić and Macuka (2018) showed that teachers who feel more fatigue, hopelessness, and anger were less engaged in their job.

Finally, it was indicated that even though both constructs (i.e., teacher self-efficacy and FLTE) exerted a significant influence on teacher work engagement, teacher self-efficacy was a stronger predictor. One possible justification regarding this finding can be the fact that teacher self-efficacy is believed to be a critical factor influencing the level of job satisfaction and teaching commitment (Fathi and Savadi Rostami, 2018). Accordingly, higher levels of job satisfaction and teaching commitment might increase a sense of confidence in the teaching ability of EFL instructors which leads to professional growth. Committed and satisfied teachers would have a strong desire for their profession and a strong attachment to it. This is confirmed by Liu (2019), who revealed that occupational self-efficacy was directly associated with organizational commitment and indirectly with work engagement *via* the mediating role of organizational commitment. Moreover, Dong and Xu’s (2022) review showed that EFL teachers’ commitment can reflect in their occupational engagement.

## Conclusion

The outcomes derived from this study suggested that although both teacher self-efficacy and FLTE predicted teacher work engagement, instructor self-efficacy had a more powerful predictive role. This is the first study that used SEM to investigate the concepts of FLTE and self-efficacy simultaneously, specifically with respect to the interconnection between these variables and work engagement. Thus, results from this research might make a unique and novel contribution to the investigations on EFL teachers’ performance. The present research adds insight to the knowledge base of teacher variables as well as teaching practices in the EFL context.

A number of implications may arise from the findings of this research. As the results of this study indicated, teacher self-efficacy was a stronger predictor of work engagement. Therefore, EFL trainers and teacher educators, institute managers, curriculum developers, and education policy makers should prioritize teacher self-efficacy in their programs and interactive workshops. Teachers might be asked to question learning suppositions and their teaching as well as establish a strong knowledge base. In order to become efficacious, EFL teachers could also (a) add learner-centered teaching approaches to their teaching practice, (b) observe the effective instruction of other teachers with similar level of proficiency and experience, and (c) use more constructive management strategies. Developing such approaches would be possible solely in an open and safe context in which teachers have flexibility in their instructional practices. School leaders and administrators are also recommended to provide EFL teachers with available teaching resources and support in this regard. Besides, EFL teachers’ affective states need to be monitored as they may affect work engagement. An initial step is that school mentors, teacher educators, and also the teachers themselves should be cognizant of the positive and negative emotions and notice that they influence their teaching quality. As for school leaders, further attention should be directed to their position as providers of emotional support and boosting/maintaining the EFL teachers’ wellbeing (e.g., foster job satisfaction and reduce stress levels). Teacher trainers at school could also pay explicit attention to EFL teachers’ emotions in their meetings. For instance, they may discuss how significant emotions could be in teacher education (e.g., discuss the effect of burnout and enjoyment on teachers’ motivation, engagement, and self-efficacy). It is consequently critical that prospective instructors enrolled in a teacher education program receive explicit support and positive messages with regard to their teaching competencies, to positively affect their teaching dedication. This helps them to realize whether they are suitable for becoming a teacher. Additionally, school psychologists could monitor and give teachers feedback on their behavior management and instructional practices.

However, while these results enhance our understanding, they should be set against available limitations. The first limitation concerns the reliance of this study on self-report measures. The utilization of self-report-only data might confine the repeatability and credibility of the results. Researchers, therefore, are encouraged to use qualitative approaches such as semi-structured interviews or observations in addition to questionnaires. Future studies could also use a mixed-methods approach for detailed examination of the link between the study variables.

In researchers’ perspective, more conclusive and precise findings in this respect will add new insights into the available literature. Nevertheless, the present results remain exploratory, and future studies validating the findings obtained is necessary, particularly in conjunction with the novel notion of FLTE and teacher engagement before they could be used to adapt professional development provisions. Also, since FLTE is still in its infancy, further empirical data and research would be welcome. Additionally, all data in this research were collected from EFL teachers in the context of Iran. This might constrain the findings from being generalized to different contexts and cultures. Future studies are also suggested examine whether the findings of the present research could be extended to different cross-cultural contexts by collecting data from other contexts to enhance the generalizability of the results. Moreover, the present results could be well served by exploring the extent to which the teacher variables generalize across different groups defined by various socio-demographic parameters, such as socio-economic background, age, gender, and level of education. Future studies could target pre-service or public-school teachers with different attitudes toward their profession. Furthermore, the Iranian EFL instructors who served as the participants of this study were selected from various educational contexts including English language institutions, high schools, and universities. Since each context has its own particularities which can affect teachers’ enjoyment, self-efficacy, and engagement, future researchers are recommended to select separate samples from various contexts (i.e., both public and private sectors) and investigate the differences among them with regard to such teacher-related variables. Further, measuring other resources (e.g., autonomy and resilience), wider factors (e.g., social support from colleagues or supervisors, organizational climate and work-related needs), and demands (e.g., job workload) may offer a new avenue for discovering how other factors influence teacher work engagement.

## Data Availability Statement

The data analyzed in this study is subject to the following licenses/restrictions: the raw data supporting the conclusions of this article will be made available by the corresponding author upon request. Requests to access these datasets should be directed to JF, jfathi13@yahoo.com.

## Ethics Statement

The studies involving human participants were reviewed and approved by University of Kurdistan. The patients/participants provided their written informed consent to participate in this study.

## Author Contributions

YX, JF, and FM were equally involved in designing the research, topic development, data collection, data analysis, writing drafts, and final editing. All authors contributed to the article and approved the submitted version.

## Conflict of Interest

The authors declare that the research was conducted in the absence of any commercial or financial relationships that could be construed as a potential conflict of interest.

## Publisher’s Note

All claims expressed in this article are solely those of the authors and do not necessarily represent those of their affiliated organizations, or those of the publisher, the editors and the reviewers. Any product that may be evaluated in this article, or claim that may be made by its manufacturer, is not guaranteed or endorsed by the publisher.
